# PyMix - The Python mixture package - a tool for clustering of heterogeneous biological data

**DOI:** 10.1186/1471-2105-11-9

**Published:** 2010-01-06

**Authors:** Benjamin Georgi, Ivan Gesteira Costa, Alexander Schliep

**Affiliations:** 1 Max Planck Institute for Molecular Genetics, Dept. of Computational Molecular Biology, Ihnestrasse 73, 14195 Berlin; 2 Center of Informatics, Federal University of Pernambuco, Recife, Brazil; 3 Dept. of Computer Science and BioMaPS Institute for Quantitative Biology, Rutgers, The State University of New Jersey, Piscataway, NJ, 08854, USA; 4 Department of Genetics, University of Pennsylvania, 528 CRB, 415 Curie Blvd PA 19104 Philadelphia, USA

## Abstract

**Background:**

Cluster analysis is an important technique for the exploratory analysis of biological data. Such data is often high-dimensional, inherently noisy and contains outliers. This makes clustering challenging. Mixtures are versatile and powerful statistical models which perform robustly for clustering in the presence of noise and have been successfully applied in a wide range of applications.

**Results:**

PyMix - the Python mixture package implements algorithms and data structures for clustering with basic and advanced mixture models. The advanced models include context-specific independence mixtures, mixtures of dependence trees and semi-supervised learning. PyMix is licenced under the *GNU General Public licence *(GPL). PyMix has been successfully used for the analysis of biological sequence, complex disease and gene expression data.

**Conclusions:**

PyMix is a useful tool for cluster analysis of biological data. Due to the general nature of the framework, PyMix can be applied to a wide range of applications and data sets.

## Background

### Clustering and biological data

The first step in the analysis of many biological data sets is the detection of mutually similar subgroups of samples by clustering. There are a number of aspects of modern, high-throughput biological data sets which make clustering challenging: The data is often high-dimensional and only a subset of the features can be expected to be informative for the purpose of an analysis. Also, the values of the data points may be distorted by noise and the data set contains a non-negligible number of missing values. In addition, many biological data sets will include outliers due to experimental artifacts. Finally, the data set might incorporate multiple sources of data from different domains (e.g different experimental methods, geno- and phenotypic data, etc.), where the relative relevance for the biological question to be addressed, as well as potential dependencies between the different sources are unknown. In particular, the latter can lead to a clustering which captures regularities not relevant to the specific biological context under consideration. Reflecting its importance in exploratory data analysis, there is a multitude of clustering methods described in the literature (see [1,2] for reviews). Clustering methods can be divided in two major groups: hierarchical and partitional methods. Hierarchical methods, which transform a distance matrix into a dendogram, have been widely used in bioinformatics, for example in the early gene expression literature, partly due to the appealing visualization of the dendograms [3]. Partitional methods are based on dividing samples into non-overlapping groups by the optimization of an objective function. For example, k-means is a iterative partitional algorithm that minimizes the sum of squared errors between samples and the centroids they have been assigned to [4].

A classical statistical framework for performing partitional clustering, which has attractive properties for biological data, are mixture models [5]. On the clustering level, due to their probabilistic nature, mixture models acknowledge the inherent ambiguity of any group assignment in exploratory biological data analysis, in a structured and theoretically sound way. This leads to a certain robustness towards noise in the data and makes mixtures a superior choice of model for data sets where hard partitioning is inappropriate. On the level of representing individual data points, mixtures are highly flexible and can adapt to a wide range of data sets and applications. Finally, there is a wealth of extensions to the basic mixture framework. For example semi-supervised learning or context-specific independence (see below for details).

In practice, the first big stepping stone for the analysis of any data set by clustering is the choice of model to be used. This can be burdensome, as most available packages are rather narrowly aimed at one specific type of model and re-implementation is time intensive. The PyMix software package aims to provide a general, high-level implementation of mixture models and the most important extensions in an object oriented setup (see additional file 1). This allows for rapid evaluation of modeling choices in an unified framework.

## Implementation

### Mixture models

Formally, a mixture model is defined as follows. Let *X *= *X*_1_,..., *X*_*p *_denote random variables (RVs) representing the features of a *p *dimensional data set *D *with *N *samples *x*_*i*_, *i *= 1,..., *N *where each *x*_*i *_consists of a realization (*x*_*i*1_,..., *x*_*ip*_) of (*X*_1_,..., *X*_*p*_). A *K *component mixture distribution is given by(1)

where the *π*_*k *_≥ 0 are the mixture coefficients with . Each of the *K *clusters is identified with one of the components. In the most straightforward case, the component distributions *P*(*x*_*i*_|*θ*_*k*_) are given by a product distribution over *X*_1_,..., *X*_*p *_parameterized by parameters *θ*_*k *_= (*θ*_*k*1_,..., *θ*_*kp*_),(2)

This is the well known naïve Bayes model (e.g. [6-8]). However, the formulation can accommodate more complex component distributions, including any multivariate distribution from the exponential family. One advantage of adopting naïve Bayes models as component disributions is that they allow the seamless integration of heterogeneous sources of data (say discrete and continuous features) in a single model. This has been made use of, for instance, for the joined analysis of gene expression and transcription factor binding data [9] or geno- and phenotype data of complex diseases [10].

When using mixtures in a clustering context, the aim is to find the assignment of data points to components which maximizes the likelihood of the data. The classical algorithm for obtaining the maximum likelihood parameters, which is also employed in PyMix, is the *Expectation Maximization *(EM) algorithm [11]. The basic idea of the EM procedure is to use the current model parameters to obtain conditional expectations of the component memberships of the data points. These expectations in turn can be used to update the model parameters in a maximum likelihood fashion. Iteration over these two steps can be shown to converge to a local maximum in the likelihood. The central quantity of EM for mixtures is the component membership posterior, i.e. the probability that a data point *x*_*i *_was generated by component *k*. By applying Bayes' rule, this posterior is given by(3)

The final cluster assignments are then also obtained based on this posterior. Each data point is assigned to the component which generated it with the highest probability, i.e. *x*_*i *_is assigned to k* = argmax_*k *_*P *(*k*|*x*_*i*_, Θ).

### Method Extensions

PyMix includes several theoretical extensions of the basic mixture framework which can be employed to adapt the method for specific applications.

#### Context-specific independence mixtures

**Figure 1:**
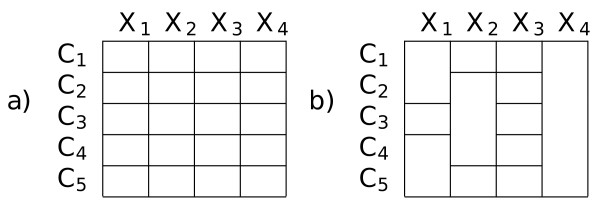
**a) Model structure for a conventional mixture with five components and four RVs**. Each cell of the matrix represents a distribution in the mixture and every RV has an unique distribution in each component. **b) **CSI model structure. Multiple components may share the same distribution for a RV as indicated by the matrix cells spanning multiple rows. In example *C*_2_, *C*_3 _and *C*_4 _share the same distribution for *X*_2_.

In the case of naïve Bayes component distributions, the component parameterization consists of a set of

parameters *θ*_*kj *_for each component *k *and feature *X*_*j*_. This can be visualized in a matrix as shown in Figure 1a). Here each cell in the matrix represent one of the *θ*_*kj*_. The different values of the parameters for each feature and component express the regularities in the data which characterize and distinguish the components. The basic idea of the *context-specific independence *(CSI) [9] extension to the mixture framework is that very often the regularities found in the data do not require a separate set of parameters for *all *features in *every *component. Rather there will be features where several component share a parameterization.

This leads to the CSI parameter matrix shown in Figure 1b). As before each cell in the matrix represents a set of parameters, but now several component might share a parameterization for a feature, as indicated by the cells spanning several rows. The CSI structure conveys a number of desirable properties to the model. First of all, it reduces the number of free parameters which have to be estimated from the data. This leads to more robust parameter estimates and reduces the risk of overfitting. Also, the CSI structure makes explicit which features characterize respectively, discriminate between clusters. In the example in Figure 1b), one can see that for feature *X*_1 _components (*C*_1_, *C*_2_) and (*C*_4_, *C*_5_) share characteristics and are represented by one set of parameters. On the other hand component *C*_3 _does not share its parameterization for *X*_1_. Moreover, if components share the same group in the CSI structure for all positions, they can be merged thus reducing the number of components in the model. Therefore learning of a CSI structure can amount to an automatic reduction of the number of components as an integral part of model training. Such a CSI structure can be inferred from the data by an application of the structural EM [12] framework. In terms of the complexity of the mixture distribution, a CSI mixture can be seen as lying in between a conventional naive Bayes mixture (i.e. a CSI structure as shown in Figure 1a) and a single naive Bayes model (i.e. the structure where the parameters of all components are identified for all features). A comparison of the performance of CSI mixtures and these two extreme cases in a classical model selection setup can be found in [13].

The basic idea of the CSI formalism to fit model complexity to the data is also shared by approaches such as *variable order Markov chains *[14] or topology learning for hidden Markov models [15]. The main difference of CSI to these approaches is that CSI identifies parameters across components (i.e. clusters) and the resulting structure therefore carries information about the regularities captured by a clustering. In this CSI mixtures bear some similarity to clustering methods such as the *shrunken nearest centroid *(SNC) algorithm [16]. However the CSI structure allows for a richer, more detailed representation of the regularities characterizing a cluster.

Another important aspect is the relation of the selection of variables implicit in the CSI structure to pure feature selection methods (e.g. [17]). These methods typically make a binary decision about the relevance or irrelevance of variables for the clustering. In contrast to that, CSI allows for more fine grained models where a variable is of relevance to distinguish subsets of clusters.

#### Dependence trees

**Figure 2:**
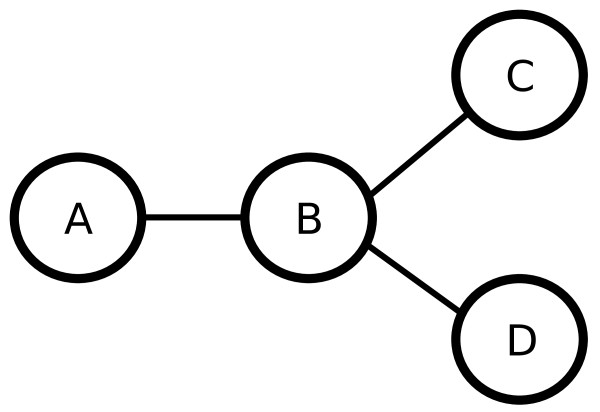
**Example of a simple DTree over features (*X*_*A*_, ***X*_*B*_, *X***_*C*_, *X*_*D*_)**. For this tree, we have the following joint distribution *P*(*x*_*A*_, *x*_*B*_, *x*_*C*_, *x*_*D*_) = *P *(*x*_*A*_)*P *(*x*_*B*_|*x*_*A*_)*P *(*x*_*C*_|*x*_*B*_)*P *(*x*_*D*_|*x*_*B*_).

The dependence tree (DTree) model extends the Naive Bayes model (Eq. 2) by assuming first-order dependencies between features. Given a directed tree, where nodes of the tree represent the features (*X*_1_,..., *X*_*p*_) and a map *pa *represents the parent relationships between features, the DTree model assumes that the distribution of feature *X*_*j *_is conditional on the feature *X*_*pa*(*j*)_. For a given tree topology defined by *pa*, the joint distribution of a DTree is defined as [18],(4)

where *P*(·|·, *θ*) is a conditional distribution, such as conditional Gaussians [19], and *θ*_*jk *_are the parameters of the conditional distribution. See Figure 2 for an example of a DTree and its distribution.

One important question is how to obtain the tree structure. For some particular applications, the structure is given by prior knowledge. In the analysis of gene expression of developmental processes for instance, the tree structure is given by the tree of cell development [20]. When the structure is unknown, the tree structure with maximum likelihood can be estimated from the data [18]. The method works by applying a maximum weight spanning tree algorithm on a fully connected, undirected graph, where vertices represent the variables and the weights of edges are equal to the mutual information between variables [18]. When the DTree models are used as components in a mixture model, a tree structure can be inferred for each component model [21]. This can be performed by applying the tree structure estimation method for each model at each EM iteration.

When conditional normals are used in Eq. 4, the DTree can be seen as a particular type of covariance matrix parameterization for a multivariate normal distribution [21]. There, the number of free parameters is linear to the number of variables, as in the case of multivariate normals with diagonal covariance matrices, while it models first-order variable dependencies. In experiments performed in [21], dependence trees compares favorably to Naive Bayes models (multivariate normal with diagonal covariance matrices) and full dependence models (multivariate normal with full covariance matrices) for finding groups of co-expressed genes and even for simulated data arising from variable dependence structures. In particular, dependence trees are not susceptible to over-fitting, which is otherwise a frequent problem in the estimation of mixture models with full diagonal matrices from sparse data.

In summary, the DTree model yields a better approximation of joint distribution in relation to the the simple Naive Bayes Model. Furthermore, the estimated structure can be useful in the indication of important dependencies between features in a cluster [21].

#### Semi-supervised learning

In classical clustering the assignments of samples to clusters is completely unknown and has to be learned from the data. This is referred to as unsupervised learning. However, in practice there is often prior information for at least a subset of samples. This is especially true for biological data, where there is often detailed expert annotation for at least some of the samples in a data set. Integrating this partial prior knowledge into the clustering can potentially greatly increase the performance of the clustering. This leads to a semi-supervised learning setup.

PyMix includes semi-supervised learning with mixtures for both hard labels as well as a formulation of prior knowledge in form of soft pairwise constraints between samples [22,23]. In this context, in addition to the data *x*_*i*_, we have a set of positive (and negative) constraints  ∈ [0, 1] ( ∈ [0, 1]), where *x*_*i*_, *x*_*j*_, 1 ≤ *i *<*j *≤ *N*. The constraints indicate a preference for pair of data points to be clustered together (positive constraints), or not clustered together (negative constraints).

**Figure 3:**
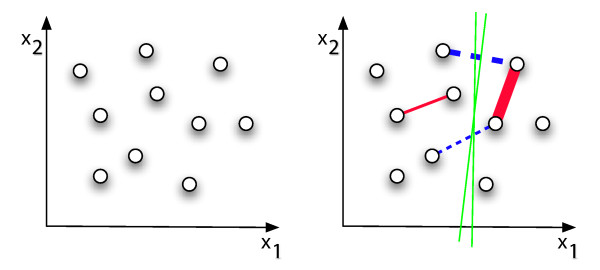
**Assuming data comes from a two-dimensional space, the addition of positive pairwise constraints, depicted as red edges, and negative constraints depicted as blue edges (right figure), support the existence of two or more clusters and indicate possible cluster boundaries (green lines)**. (Figure reproduced from [29])

The main idea behind the semi-supervised method implemented in Pymix is to find a clustering solution *Y *where the least number of constraints are violated [22] (see Figure 3). This can be achieved by redefining the posterior assignment rule of the EM (Eq. 3), as

where *r*_*jk *_= *P *(*k*|*x*_*j*_, Θ, *W*), *W *is the set of all positive and negative constraints and (*λ*^+^, *λ*^-^) are Lagrange parameters defining the penalty weights of positive and negative constraint violations.

Hard labels arise as a special case of the above, when the constraints are binary ( ∈ {0, 1},  ∈ {0, 1}) such that there is no overlap in the constraints, and the penalty parameters are set to high values *λ*^+ ^= *λ*^-^~∞. In this scenario, only solutions in full accordance with the constraints are possible [23].

There are several semi-supervised/constraint-based clustering methods described in the literature [24]. We choose to implement the method proposed in [22], because it can be used within the mixture model framework and supports the use of soft-constraints. Therefore, we can take simultaneous advantage of the characteristics of the probability distribution offered in Pymix and the semi-supervised framework. Moreover, the soft-constraints allow for the inclusion of our prior believes into the constraints, an important aspect in error prone biological data.

### Dependencies

PyMix is written in the *Python *programming language http://www.python.org. It includes a custom written C extension and makes use of the numpy http://numpy.scipy.org array package, the GNU Scientific library (GSL) http://www.gnu.org/software/gsl/ and matplotlib http://matplotlib.sourceforge.net plotting capabilities.

### Overall architecture

**Figure 4:**
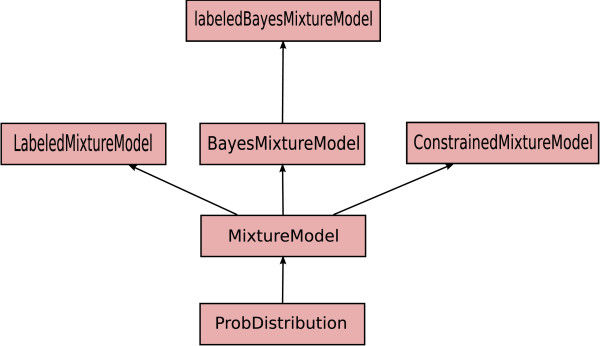
**Object hierarchy of the MixtureModel class**. MixtureModel is derived from the base class Prob-Distribution and all the specialized mixture model variant classes are derived from it.

PyMix represents finite mixture models in an object oriented framework. Figure 4 shows the class tree for the MixtureModel class. MixtureModel represents the standard finite mixture model. All the extensions to the mixture framework implemented in PyMix mentioned previously, are derived as specialized classes from MixtureModel. The CSI structure learning is implemented as part of BayesMixtureModel.

LabeledMixtureModel and ConstrainedMixtureModel represent two different approaches to semi-supervised learning. The former implements semi-supervised learning with hard labels, the latter with pairwise constraints on the data points. Full documentation of the objects hierarchy in PyMix (including the dependence trees) can be found on the PyMix website http://www.pymix.org.

Currently, the framework supports mixtures of Gaussians, discrete distributions and exponential distributions. Furthermore, the framework has an extension that allows hidden Markov models as components by the use of the GHMM library http://www.ghmm.org and is also used by the GQL tool [25]. Moreover, due to the object oriented setup, it is easily extendable and more distributions will be added in the future.

### Prior work

Due to the popularity and versatility of the mixture approach, there is a considerable number of software packages available. For instance the R package MCLUST http://www.stat.washington.edu/mclust/ implements algorithms for Gaussian mixture estimation. The MIXMOD http://www-math.univ-fcomte.fr/mixmod/ C++ package contains algorithms for conventional mixtures of Gaussian and multinomial distributions with MATLAB bindings. Another R package for conventional mixture analysis is the MIX http://icarus.math.mcmaster.ca/peter/mix/mix.html package. An example for a rather specialized package would be Mtreemix http://mtreemix.bioinf.mpi-sb.mpg.de/ which allows estimation of mixtures of mutagenic trees. In general it can be said that most of these packages focus rather narrowly on specific model types. The main advantage of PyMix is that the general, object oriented approach allows for a wide variety of mixture variants to be integrated in a single, unified framework. The different advanced models (CSI or semi-supervised) and component distributions (e.g. dependence trees) available in PyMix, make the software applicable for many applications. Also, the object orientation means that the software can be straightforwardly extended with additional model types by advanced users.

## Results and Discussion

### PyMix example session

Assume we have a data set of DNA sequences of length ten and we would like to perform clustering with a standard mixture model. The data is stored in a fasta-file format 'dataset.fa'.

After starting the Python interpreter we first import the required PyMix modules.

   >>> import mixture, bioMixture

Here, mixture is the main PyMix module, bioMixture contains convenience functions for the work with DNA and protein sequences.

The next step is to read in the data from the flat file.

   >>> data = bioMixture.readFastaSequences('dataset.fa')

The function readFastaSequences parses the sequences in dataset.fa and returns a new DataSet object. Now that the data is available we set up the model and perform parameter estimation using the EM algorithm.

   >>> m = bioMixture.getModel(2,10)

   >>> m.EM(data,40,0.1)

   Parsing data set.done

   Step 1: log likelihood: -130.1282276 (diff = -129.1282276)

   Step 2: log likelihood: -40.1031405877 (diff = 90.0250870124)

   Step 3: log likelihood: -39.1945767199 (diff = 0.908563867739)

   Step 4: log likelihood: -37.8237645332 (diff = 1.37081218671)

   Step 5: log likelihood: -36.2537338607 (diff = 1.5700306725)

   Step 6: log likelihood: -33.9000749475 (diff = 2.35365891327)

   Step 7: log likelihood: -31.9680428475 (diff = 1.93203209999)

   Step 8: log likelihood: -31.6274670433 (diff = 0.340575804189)

   Step 8: log likelihood: -31.6079141237 (diff = 0.0195529196039)

   Convergence reached with log_p -31.6079141237 after 8 steps.

The first argument to getModel is the number of clusters (2 in the example), the second gives the length of the sequences (10 in this case). The EM function takes a DataSet as input and performs parameter estimation. The second and third argument are the maximum number of iterations and the convergence criterion respectively. The output shows that in the exmaple the EM took 8 iterations to converge. Note that in practice one would perform multiple EM runs from different starting points to avoid local maxima. This is implemented in the randMaxEM function.

Finally, we perform cluster assignment of the sequences.

   >>> c = m.classify(data)

   classify loglikelihood: -31.6079141237.

   ** Clustering **

   Cluster 0, size 4

   ['seq24', 'seq33', 'seq34', 'seq36']

   Cluster 1, size 6

   ['seq10', 'seq21', 'seq28', 'seq29', 'seq30', 'seq31']

   Unassigend due to entropy cutoff:

   []

The classify function returns a list with the cluster label of each sequence (and prints out the clustering). This list can now be used to perform subsequent analysis'. PyMix offers various functionalities for visualization or printing of clusterings, ranking of features by relevance for the clustering or cluster validation. This and other examples for working with PyMix can be found in the *examples *directory in the PyMix release. Documentation for the PyMix library and a more detailed tutorials can be found on the PyMix website http://www.pymix.org.

### PyMix applications

PyMix has been applied on clustering problems in a variety of biological settings. In the following we give a short description of some these applications. For more details we refer to the original publications.

#### Context-specific independence mixtures

The CSI structure gives an explicit, high level overview of the regularities that characterize each cluster. This greatly facilitates the interpretation of the clustering results. For instance the CSI formalism has been made use of for the analysis of transcription factor binding sites [13]. There, the underlying biological question under investigation was whether there are transcription factors which have several, distinct patterns of binding in the known binding sites. In this study CSI mixtures were found to outperform conventional mixture models and biological evidence for factors with complex binding behavior were found.

**Figure 5:**
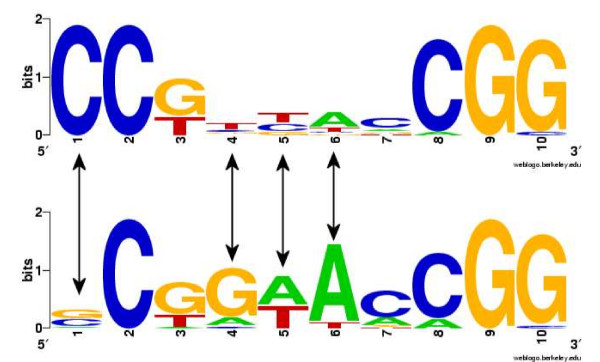
**WebLogos http://weblogo.berkeley.edu for the two subgroups of Leu3 binding sites**. It can be seen that the positions with strong sequence variability (positions 1, 4, 5 and 6) have been recognized by the CSI structure (indicated by arrows). (Figure reproduced from [13].)

An example for the two clusters of binding sites found for the transcription factor Leu3 is shown in Figure 5. The double arrows indicate the positions where the learned CSI structure assigned a separate distribution for each cluster. These positions coincide with the strongest difference in the sequence composition of the two clusters. In a comparison on a data set of 64 JASPAR [26] transcription factors, the CSI mixtures outperformed conventional mixtures and positional weight matrices with respect to human-mouse sequence conservation of predicted hits [13].

Another application of CSI mixtures was the clustering of protein subfamily sequences with simultaneous prediction of functional residues [27]. Here, the CSI structure was made use of to find residues which differed strongly and consistently between the clusters. Combined with a ranking score for the residues, this allowed the prediction of putative functional residues. The method was evaluated with favorable results on several data sets of protein sequences.

Finally, CSI mixture were brought to bear on the elucidation of complex disease subtypes on a data set of attention deficit hyperactivity (ADHD) phenotypes [10]. The clustering and subsequent analysis of the CSI structure revealed interesting patterns of phenotype characteristics for the different clusters found.

#### Dependence trees

The dependence tree distribution allows modeling of first order dependencies between features. In experiments performed with simulated data [21], dependence trees compared favorably to naive Bayes and full dependence models for finding groups arising from variable dependence structures. In particular, dependence trees are not susceptible to over-fitting, which is otherwise a frequent problem in the estimation of mixture models from sparse data. Thus, it offers a better approximation of the joint distribution in relation to the the simple Naive Bayes Model.

**Figure 6:**
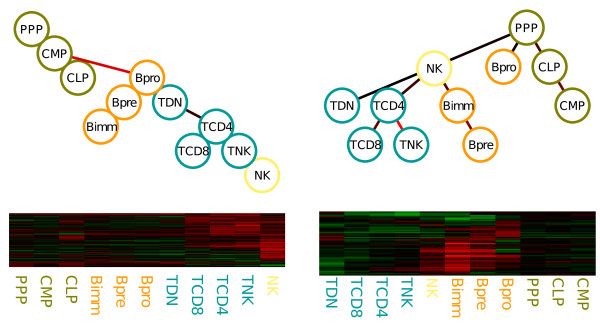
**We depict the dependence trees and the expression patterns of 2 groups of a Lymphoid development data **[21]. Expression profiles are indicated as a heat-map, where red values indicate over-expression and green values indicate under-expression. Lines correspond to genes and columns correspond to the developmental stage ordered as in the corresponding dependence tree. In the left cluster, genes have a over-expression patterns for T cell related stages (stages in blue); while the cluster in the right, we have over-expression of B cell related stages (stages in orange). The dependence tree of each cluster reflects the co-expression of developmental stages within the clusters.

One particular application is the analysis of patterns of gene expression in the distinct stages of a developmental tree, the developmental profiles of genes. It is assumed that, in development, the sequence of changes from a stem cell to a particular mature cell, as described by a developmental tree, are the most important in modeling gene expression from developmental processes. For example in [21], we analyzed a gene expression compendia of Lymphoid development, which contained expression from lymphoid stem cells, B cells, T cells and Natural Killer cells (depicted in green, orange, blue and yellow respectively in Figure 6).

By combining the methods for mixture estimation and for the inference of the DTree structure, it is possible to find DTree dependence structure specific to groups of co-regulated genes (see Figure 6). In the left cluster, genes have a over-expression patterns for T cell developmental stages (in blue); while in the right cluster, we have over-expression of B cell developmental stages (stages in orange). There, the estimated structure indicates important dependencies between features (developmental stages) in a cluster. We also carried out a comparative analysis, where mixtures of DTrees had a higher recovery of groups of genes participating in the same biological pathways than other methods, such as normal mixture models, k-means and SOM.

#### Semi-supervised learning

Semi-supervised learning can improve clustering performance for data sets where there is at least some prior knowledge on either cluster memberships or relations between samples in the data.

An example for the former has been investigated in the context of protein subfamily clustering [28]. There, the impact of differing amounts of labeled samples on the clustering of protein subfamilies is investigated. For several data sets of protein sequences the impact and practical implications of the semi-supervised setup are discussed. In [23], the impact of adding a few high quality constraints for identifying cell cycle genes in data from gene expression time courses with a mixture of hidden Markov models was demonstrated.

**Figure 7:**
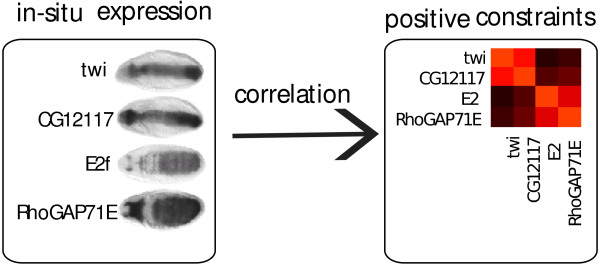
**Example of positive constraints used for syn-expression study **[29]. For a set of genes, we estimate the correlation between the image intensities, and use the correlation matrix as positive constraints. High correlations (red entries in the constraint matrix) indicate expression co-location. use these to constraints the clustering o gene expression time-courses, for finding genes which are co-expressed

If there is prior knowledge on the relations of pairs of samples, this can be expressed in form of pairwise must-link or must-not-link constraints. This leads to another variant of semi-supervised learning. One application of this framework was the clustering of gene expression time course profiles and in-situ RNA hybridization images of drosophila embryos [29]. There, the constraints were obtained by measuring the correlation between in-situ RNA hybridization images of genes pairs (see Figure 7). These constraints differentiate between genes showing co-expression only by chance from those temporal co-expression supported by spatial co-expression (syn-expression). It could be shown that with the inclusion of few high quality soft constraints derived from in-situ data, there was an improvement in the detection of syn-expressed genes [29].

## Conclusions

PyMix-the Python mixture package is a powerful tool for the analysis of biological data with basic and advanced mixture models. Due to the general formulation of the framework, it can be readily adapted and extended for a wide variety of applications. This has been demonstrated in multiple publications dealing with a wide variety of biological applications.

## Availability and requirements

**Project name**: PyMix - the Python mixture package

**Project home page**: http://www.pymix.org

**Operating system(s)**: Platform independent

**Programming language**: Python, C

**Other requirements**: GSL, numpy, matplotlib

**License **GPL

## Authors' contributions

BG is the main PyMix developer. IC contributed code for the semi-supervised learning and the mixtures of dependence trees. AS provided supervision and guidance. All authors have contributed to the manuscript.

## Supplementary Material

Additional file 1 **Pymix release 0.8a**. Sources for the latest Pymix release. For the most recent version visit http://www.pymix.org. Click here for file
